# Performance Evaluation of Triple-Frequency GPS/Galileo Techniques for Precise Static and Kinematic Applications

**DOI:** 10.3390/s21103396

**Published:** 2021-05-13

**Authors:** Mahmoud Abd Rabbou, Mohamed Abdelazeem, Salem Morsy

**Affiliations:** 1 Public Works Department, Faculty of Engineering, Cairo University, 12613 Giza, Egypt; mahmoud.abdelrahman@cu.edu.eg; 2 Civil Engineering Department, Faculty of Engineering, Aswan University, 81542 Aswan, Egypt; moh.azm84@gmail.com

**Keywords:** triple-frequency, semi-decoupled, between-satellite single-difference (BSSD), GPS/Galileo, precise point positioning (PPP)

## Abstract

The objective of this research was to develop new precise point positioning (PPP) processing models using triple-frequency GPS/Galileo observations. Different triple-frequency PPP models were developed including undifferenced, between-satellite single-difference (BSSD) and semi-decoupled PPP models. Additionally, a dual-frequency ionosphere-free undifferenced PPP model was developed. The performance of our developed PPP models was evaluated for both static and kinematic applications. To validate the proposed PPP models for static applications, triple-frequency GPS/Galileo observations spanning three successive days from eight globally distributed reference stations were acquired. Then, the observations were processed using the four static PPP solutions. It is found that the 3D positioning accuracy of the triple-frequency semi-decoupled, BSSD and undifferenced PPP models is enhanced after 10 min by about 50, 41 and 29%, respectively, compared with the dual-frequency undifferenced PPP model. After 20 min of processing, improvements in the 3D positioning accuracy by 40, 31 and 21% are obtained for the triple-frequency semi-decoupled, BSSD and undifferenced PPP models, respectively, with respect to the dual-frequency PPP model. The 3D positioning accuracy is also improved after 60 min, compared with the dual-frequency solution, by 40, 40 and 35% for the triple-frequency semi-decoupled, BSSD and undifferenced PPP solutions, respectively. For kinematic application validation, a vehicle trajectory was carried out. The collected triple-frequency GPS/Galileo observations were processed using the four kinematic PPP solutions. It is shown that the triple-frequency semi-decupled, BSSD and undifferenced PPP solutions enhance the 3D positioning accuracy by 31, 23 and 10%, respectively, in comparison with the dual-frequency undifferenced PPP solutions.

## 1. Introduction

The precise point positioning (PPP) technique using dual-frequency Global Positioning System (GPS) observations provides centimeter- and decimeter-level positioning accuracy for static and kinematic modes, respectively [1,2]. The PPP positioning accuracy is based on the quality of observations, satellite availability and geometry and carrier phase ambiguity parameters. The PPP technique can be used in a number of precise applications. Examples of these applications include precise surveying, hydrographic survey, disaster monitoring, atmospheric sounding and space weather applications [3,4,5,6,7]. Unfortunately, however, the main limitation of the PPP solution is that it needs a long time to converge to centimeter or decimeter positioning accuracy. This is attributed to the satellite geometry, inappropriate modeling of receiver and satellite biases and integer ambiguity resolution.

The satellite availability and geometry can be improved by using multi-constellation global navigation satellite system (GNSS) observations such as GLONASS, Galileo and BeiDou. The addition of multi-GNSS observations increases the measurement redundancy, which in turn accelerates the convergence time and enhances the positioning accuracy. However, it introduces additional unknowns in the parameter estimation procedure such as inter-system bias (ISB). Inter-system bias can be defined as the difference between the receiver differential code bias (DCB) of the GPS and other GNSS satellites.

A number of researchers have investigated the performance of multi-GNSS PPP solutions, e.g., [8,9,10,11,12,13,14,15,16,17]. Cai et al. [11] developed a quad-constellation PPP model for processing both static and kinematic observations. The performance of the developed PPP model was evaluated in terms of positioning accuracy and convergence time. It was shown that the GPS/BeiDou PPP model improved both the positioning accuracy and convergence time in three coordinate components in comparison with the GPS-only PPP model. In addition, the performance of the GPS/GLONASS PPP model was superior to the GPS/BeiDou PPP model. The positioning accuracy of the triple GPS/GLONASS/BeiDou PPP model was significantly improved in comparison with the dual-constellation PPP model. The accuracy of the PPP model was slightly enhanced after adding Galileo observations. Kiliszek and Kroszczyński [14] evaluated the performance of the triple GPS/GLONASS/Galileo PPP solution over three different periods in 2017, 2018 and 2019 using different cut-off elevation angle values. It was found that the accuracy of the GPS/GLONASS/Galileo PPP solution was significantly improved in 2019. This was mainly because the Galileo system was further developed and more visible satellites were available in 2019. It was also shown that 90% of solution availability with centimeter-level positioning accuracy was achieved using triple GPS/GLONASS/Galileo observations with a 40° cut-off elevation angle.

To speed up the PPP convergence time, both the satellite- and receiver-related biases should be modeled. Satellite-related biases are relatively stable over time, while receiver-related biases vary with time. In addition, satellite-related biases are lumped into the ambiguity parameters along with receiver-related biases, affecting the ambiguity nature. The between-satellite single-difference (BSSD) ionosphere-free PPP technique can be used in order to cancel out the receiver-related biases from both code and phase observations. The BSSD technique can be used with two different combinations, namely, the loose BSSD and the tight BSSD. For the loose BSSD combination, multiple GNSS satellites are used as reference satellites by taking into account that a minimum of two satellites from each single GNSS system should be available. For the tight BSSD combination, a GPS satellite is used as a reference for other satellite systems. However, the ISBs are introduced here as additional unknowns.

The BSSD technique has been investigated by a number of researchers using different scenarios, e.g., [18,19,20,21,22,23,24,25,26]. Abd Rabbou and El-Rabbany [18] developed undifferenced and BSSD ionosphere-free PPP models using multi-GNSS observations. It was found that the positioning accuracy of the BSSD PPP model was superior to that of the undifferenced PPP model. The accuracy of the GPS/Galileo BSSD PPP model was enhanced by about 30%, 27% and 10% for latitude, longitude and altitude components, respectively, with respect to the undifferenced PPP model. In addition, the accuracy of the GPS/BeiDou BSSD PPP model was improved by about 17%, 22% and 15% for latitude, longitude and altitude, respectively, while the accuracy of the multi-GNSS model was improved by about by 22% and 15% in latitude and altitude, respectively, with respect to the traditional undifferenced PPP model. Moreover, the convergence time of the BSSD PPP model was enhanced in comparison with the undifferenced PPP model. Afifi and El-Rabbany [20] investigated the performance of the GPS/Galileo BSSD PPP model using both loose and tight combinations in comparison with the traditional undifferenced GPS-only PPP model. Static datasets for a number of reference stations over a period of six days were processed. It was shown that the positioning accuracy of the GPS/Galileo BSSD PPP model was improved by about 50% and 25% for the loose and tight combinations, respectively, in comparison with the undifferenced GPS-only PPP model. Moreover, the convergence time of the GPS/Galileo BSSD PPP solution was accelerated by about 50% for both combinations in comparison with the undifferenced GPS-only PPP solution.

To further improve the PPP solution convergence time, the ambiguity parameters should be resolved. A number of PPP integer ambiguity resolution techniques have been developed, e.g., [27,28,29,30,31,32,33,34]. The ambiguity resolution can be divided into three main techniques, which are the fractional cycle bias (FCB), the integer recovery clock (IRC) and the decoupled clock (DC) techniques. Collins et al. [27] developed the PPP decoupled clock ambiguity resolution technique by separating the code and phase receiver clock parameters by introducing a new phase receiver clock. In this model, the carrier phase ambiguity was isolated to be an integer value using a fixed ambiguity datum. A network solution was required in order to estimate the satellite code and phase clock parameters and then use these parameters in the PPP solution. The decoupled clock method was further developed in order to include multi-GNSS observations, namely, the multiple ambiguity datum (MAD) PPP method [32]. For the MAD PPP method, a multiple ambiguity datum was used as multiple receiver clock differences existed.

Currently, triple-frequency observations are available from the modernized GPS, BeiDou and Galileo systems. The availability of triple-frequency observations provides measurement redundancy, different linear combinations and fast ambiguity resolution, which in turn improves the PPP solution. Unlike dual-frequency PPP, additional unknown parameters are introduced including phase and code inter-frequency biases (IFBs) for both receivers and satellites. Receiver IFBs can be removed through the BSSD technique. Satellite phase inter-frequency bias, known as inter-frequency clock bias (IFCB), is the difference between the estimated clock from L1/L2 and the estimated clock from L1/L5 combinations. On the other hand, satellite code IFB is the difference between the computed DCBP1-P2 and the computed DCBP1-P5. Satellite phase IFB is time-dependent, while satellite code IFB is constant [35,36].

More recently, the performance of triple-frequency PPP has been examined in a number of studies, e.g., [37,38,39,40,41,42,43,44,45,46]. Li et al. [42] developed a triple-frequency triple-constellation (i.e., GPS, BeiDou and Galileo) precise point positioning ambiguity resolution (PPP-AR) model. Their developed model was validated using static and kinematic datasets. It was found that the triple-frequency PPP-AR model made a significant contribution to the convergence time with respect to the dual-frequency PPP-AR model for both static and kinematic datasets. In addition, the triple-frequency PPP-AR model had a marginal effect on the positioning accuracy compared with the dual-frequency PPP-AR model. Pan et al. [44] compared between three widely used triple-frequency PPP solutions, namely, PPP using L1/L2 and L2/L5 dual-frequency ionosphere-free linear combinations (IF-PPP1), PPP using a triple-frequency ionosphere-free linear combination (IF-PPP2) and PPP using uncombined (UC) observations (UC-PPP). Static GPS observations were used. It was shown that the positioning accuracy of the three models was comparable. For the convergence time, IF-PPP2 was superior to the other two models.

Our study aims to develop triple-frequency GPS/Galileo PPP models using the undifferenced, BSSD and semi-decoupled techniques. A dual-frequency ionosphere-free PPP model is developed as well. Static triple-frequency GPS/Galileo measurements from a number of reference stations are used in order to assess the performance of the developed PPP models for static positioning applications. In addition, triple-frequency GPS/Galileo observations are collected from a moving vehicle and then processed to investigate the proposed PPP solutions’ accuracy for kinematic applications.

## 2. Basic GNSS Observation Equations

The most common GNSS observation equations for both the pseudorange and carrier phase observables can be written as follows [47]:

(1) $$P_{i}^{j}=\rho^{j}+cdt_{r,j}-c\;dt^{j}+\frac{f_{i}^{2}}{f_{o}^{2}}I_{i}^{j}+T^{j}+e_{i}^{j}$$

(2) $$\Phi_{i}^{j}=\rho^{j}+cdt_{r,j}-c\;dt^{j}-\frac{f_{i}^{2}}{f_{o}^{2}}I_{i}^{j}+T^{j}+\lambda_{i}N_{i}+\varepsilon_{i}^{j}$$

where i, j and r refer to the frequency, GNSS satellite and receiver, respectively; P and Φ are pseudorange and carrier phase measurements; $\rho^{j}$ is the satellite–receiver true geometric range; c is the speed of light in vacuum; $dt_{r,j}$ and $\;dt^{j}$ refer to the clock errors for the receiver and satellite, respectively; $f_{i}$ is the carrier phase frequency; $f_{o}$ is the base GNSS frequency ($f_{o}$ = 10.23 MHz); $I_{i}^{j}$ denotes the ionospheric delay; $T^{j}$ is the tropospheric delay; $\lambda_{i}$ is the wavelength of the carrier phase; $N_{i}\;$ is the non-integer ambiguity parameter; $e_{i}^{j}$ and $\varepsilon_{i}^{j}$ are the code and carrier phase unmodeled residual errors, respectively.

## 3. Triple-Frequency PPP Model

The triple-frequency linear combination for both pseudorange and carrier phase observations can be formed as follows [37]:

(3) $$P_{3}=\alpha_{1}P_{1}+\alpha_{2}P_{2}+\alpha_{3}P_{3}$$

(4) $$\Phi_{3}=\alpha_{1}\Phi_{1}+\alpha_{2}\Phi_{2}+\alpha_{3}\Phi_{3}$$

where $\alpha_{1},\;\alpha_{2}$ and $\alpha_{3}$ are the coefficients of the linear combination of the three frequencies’ ($f_{1}$, $f_{2}$ and $f_{3}$) observations. The geometric constraint can be achieved as follows [38]:

(5) $$\alpha_{1}+\alpha_{2}+\alpha_{3}=1$$

The ionosphere-free linear combination is used in order to eliminate the ionospheric delay for dual-frequency observations. For the triple-frequency observations, however, the ionospheric delay can be eliminated using the following form [40]:

(6) $$\frac{\alpha_{1}}{f_{1}^{2}}+\frac{\alpha_{2}}{f_{2}^{2}}+\frac{\alpha_{3}}{f_{3}^{2}}=0$$

To estimate the noise of the triple-frequency combination, its value is assumed to be the same for the three carrier phases, and there is no correlation between them. Thus, the noise of the combined observable can be mathematically expressed as follows [40]:

(7) $$\sigma_{c}^{2}=\sigma_{\Phi i}^{2}\left(\alpha_{1}^{2}+\alpha_{2}^{2}+\alpha_{3}^{2}\right)=\sigma_{\Phi i}^{2}\epsilon^{2}$$

where $\;\sigma_{c}$ is the noise of the combined observable; $\sigma_{\Phi i}$ is the standard deviation of the carrier phases on frequency i; ϵ refers to the noise amplification factor. The amplification factor is high for the dual-frequency ionosphere-free PPP model. However, for triple-frequency PPP, the implication factor can be minimized using the following form:

(8) $$\alpha_{1}^{2}+\alpha_{2}^{2}+\alpha_{3}^{2}=\epsilon^{2}=min$$

### 3.1. Undifferenced Triple-Frequency GPS/Galileo PPP Model

The undifferenced triple-frequency PPP model using GPS and Galileo observations can be written using Equations (1) and (2) as follows [46]:

(9) $$P_{Tun}^{G}=\rho^{G}+cdt_{r,G}-c\;dt^{G}+T_{w}^{G}+e^{G}$$

(10) $$\Phi_{Tun}^{G}=\rho^{G}+cdt_{r,G}-c\;dt^{G}+T_{w}^{G}+{\left(\lambda N\right)}_{T}^{G}+\varepsilon^{G}$$

(11) $$P_{Tun}^{E}=\rho^{E}+cdt_{r,G}-c\;dt^{E}+T_{w}^{E}+ISB_{E}+e^{E}$$

(12) $$\Phi_{Tun}^{E}=\rho^{E}+cdt_{r,G}-c\;dt^{E}+T_{w}^{E}+{\left(\lambda N\right)}_{T}^{E}+ISB_{E}+\varepsilon^{E}$$

where G and E refer to GPS and Galileo satellite systems, respectively; $P_{Tun}^{G}$ and $P_{Tun}^{E}$ are the undifferenced triple-frequency pseudoranges combination for GPS and Galileo systems, respectively; $\Phi_{Tun}^{G}$ and $\Phi_{Tun}^{E}$ are the undifferenced triple-frequency carrier phases combination for GPS and Galileo systems, respectively; $T_{w}^{G}$ and $T_{w}^{E}$ are the wet zenith tropospheric delays for GPS and Galileo, respectively; $ISB_{E}$ is the inter-system bias.

### 3.2. Between-Satellite Single-Difference Triple-Frequency GPS/Galileo PPP Model

To completely eliminate the receiver-related biases for both pseudorange and carrier phase measurements, the BSSD PPP technique can be used for the triple-frequency combination model. The mathematical expression for the triple-frequency BSSD GPS/Galileo PPP model takes the following form [18]:

(13) $$P_{Tbssd}^{G}=∆\rho^{G}-∆c\;dt^{G}+∆T_{w}^{G}+∆e^{G}$$

(14) $$\Phi_{Tbssd}^{G}=∆\rho^{G}-∆c\;dt^{G}+∆T_{w}^{G}+∆{\left(\lambda N\right)}_{T}^{G}+∆\varepsilon^{G}$$

(15) $$P_{Tbssd}^{E}=∆\rho^{E}-∆c\;dt^{E}+∆T_{w}^{E}+∆e^{E}$$

(16) $$\Phi_{Tbssd}^{E}=\rho^{E}-∆c\;dt^{E}+∆T_{w}^{E}+∆{\left(\lambda N\right)}_{T}^{E}+∆\varepsilon^{E}$$

where $P_{Tbssd}^{G}$ and $P_{Tbssd}^{E}$ are the triple-frequency BSSD for GPS and Galileo pseudorange observables, respectively; $\Phi_{Tbssd}^{G}$ and $\Phi_{Tbssd}^{E}$ are the triple-frequency BSSD for GPS and Galileo carrier phase observables, respectively. As given in Equations (13)–(16), the triple-frequency receiver pseudorange and carrier phase biases are removed. It should be mentioned that the tight BSSD combination is applied in our developed model.

### 3.3. Triple-Frequency Semi-Decoupled GPS/Galileo PPP Model

The semi-decoupled method can be used in order to separate the code and phase receiver clock parameters. To apply this method, one of the satellite ambiguities is fixed by an arbitrary value. Then, the combined receiver phase clock and the fixed satellite ambiguity are considered to be a reference receiver phase clock for other receiver phase clocks. For the triple-frequency GPS/Galileo combination model, multiple reference satellites are selected in which the receiver phase clock is shifted by the fixed ambiguity of the GPS or Galileo satellite. The mathematical model of the triple-frequency semi-decoupled GPS/Galileo PPP model can be expressed as follows [20]:

(17) $$\Phi_{Tsd}^{G1}=\rho^{G}+\left[cdt_{r,G}^{\Phi }+{\left(\lambda N\right)}_{T}^{G1}\right]-c\;dt^{\Phi ,G1}+T_{w}^{G1}+\varepsilon^{G}$$

(18) $$\Phi_{Tsd}^{G2}=\rho^{G}+\left[cdt_{r,G}^{\Phi }+{\left(\lambda N\right)}_{T}^{G1}\right]-c\;dt^{\Phi ,G2}+T_{w}^{G2}+\left[{\left(\lambda N\right)}_{T}^{G2}-{\left(\lambda N\right)}_{T}^{G1}\right]+\varepsilon^{G}$$

(19) $$\Phi_{Tsd}^{E1}=\rho^{E}+\left[cdt_{r,G}^{\Phi }+{\left(\lambda N\right)}_{T}^{G1}\right]-c\;dt^{\Phi ,E1}+T_{w}^{E1}+\left[ISB_{E}+{\left(\lambda N\right)}_{T}^{E1}-{\left(\lambda N\right)}_{T}^{G1}\right]+\varepsilon^{E}$$

(20) $$\Phi_{Tsd}^{E2}=\rho^{E}+\left[cdt_{r,G}^{\Phi }+(\lambda N{)}_{T}^{G1}\right]-cdt^{\Phi ,E2}+T_{w}^{E2}+\left[ISB_{E}+(\lambda N{)}_{T}^{E1}-(\lambda N{)}_{T}^{G1}\right]+\left[(\lambda N{)}_{T}^{E2}-(\lambda N{)}_{T}^{E1}\right]+\varepsilon^{E}$$

where $\Phi_{Tsd}^{G}$ and $\Phi_{Tsd}^{E}$ are the triple-frequency semi-decoupled phase observables for GPS and Galileo, respectively; 1 and 2 refer to the satellite numbers, where 1 is the reference satellite; $dt_{r,G}^{\Phi }$ is the phase receiver clock parameters; $dt^{\Phi ,G}$ and $\;dt^{\Phi ,E}$ are the phase satellite clocks for GPS and Galileo systems, respectively.

In the T-SD PPP model, a new phase receiver clock is introduced (i.e., $\left[cdt_{r,G}^{\Phi }+{\left(\lambda N\right)}_{T}^{G1\;}\right]$). Thus, only one phase receiver clock parameter is computed. The carrier phase ambiguities for the remaining satellites are the difference between the satellite ambiguity and the reference satellite ambiguity for both GPS and Galileo satellite systems. In addition, the triple-frequency inter-system bias of the remaining Galileo satellites is eliminated.

## 4. GPS/Galileo Datasets Processing

**Figure 1:**
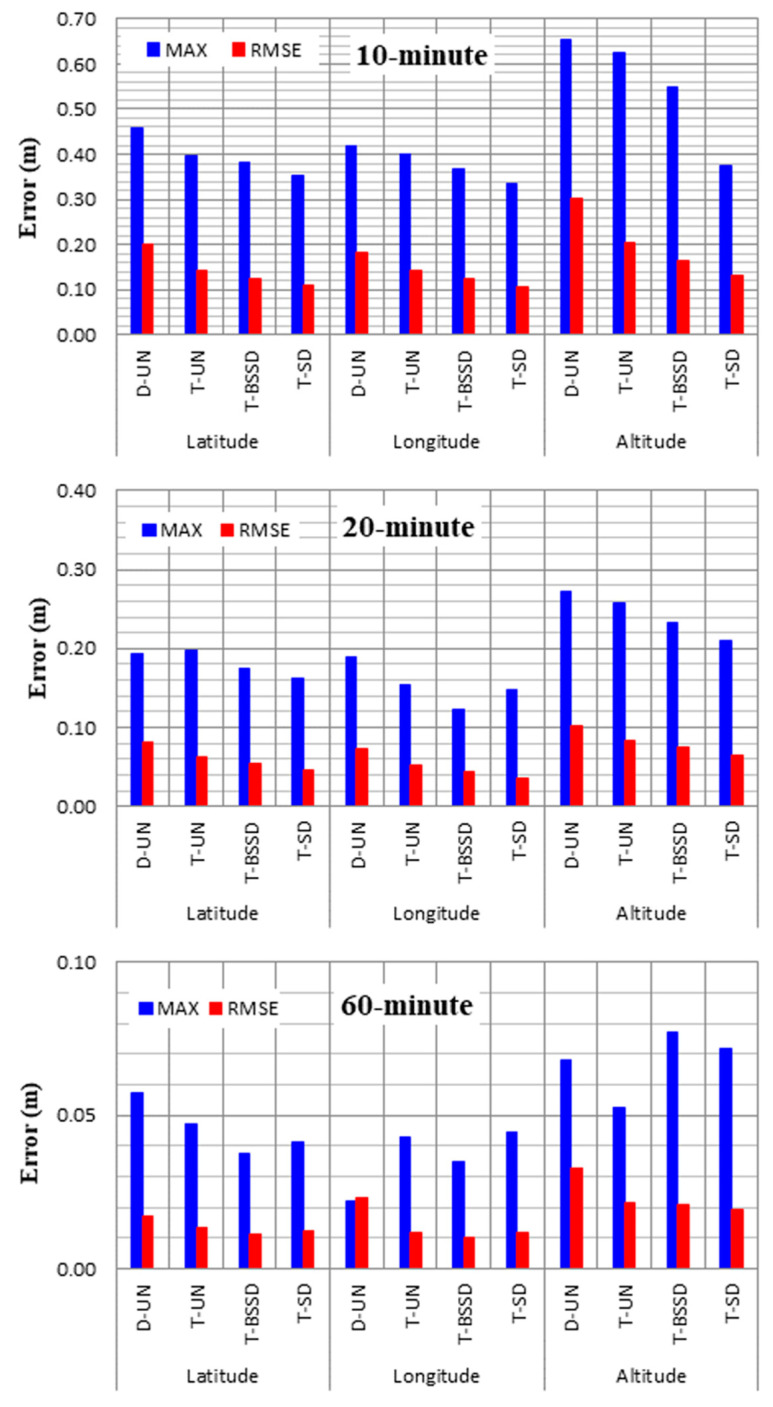
Distribution of the examined stations.

Our developed triple-frequency PPP models were validated for static and kinematic applications. Static datasets from eight international GNSS service (IGS) multi-GNSS experiment (MGEX) reference stations were used. The examined stations were selected on a global scale, as shown in Table 1. Figure 1 illustrates the spatial distribution of the examined stations. Triple-frequency GPS/Galileo observations spanning 24 h over three consecutive days (i.e., day of year (DOY) 1, 2 and 3 in 2019) were downloaded [48]. Then, the observations were processed using the developed triple-frequency GPS/Galileo PPP models. Additionally, the traditional dual-frequency ionosphere-free undifferenced PPP solution was used.

**Figure 2:**
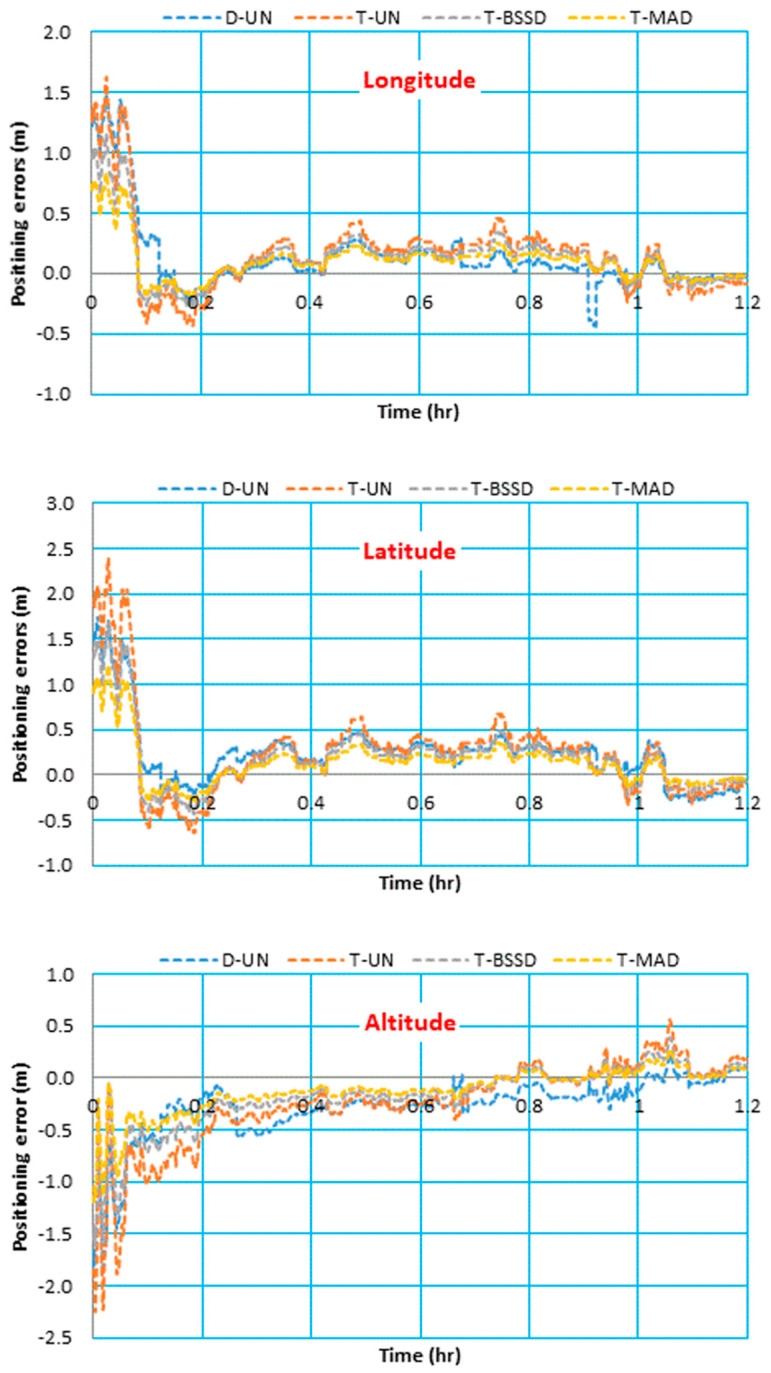
Vehicle trajectory.

**Figure 3:**
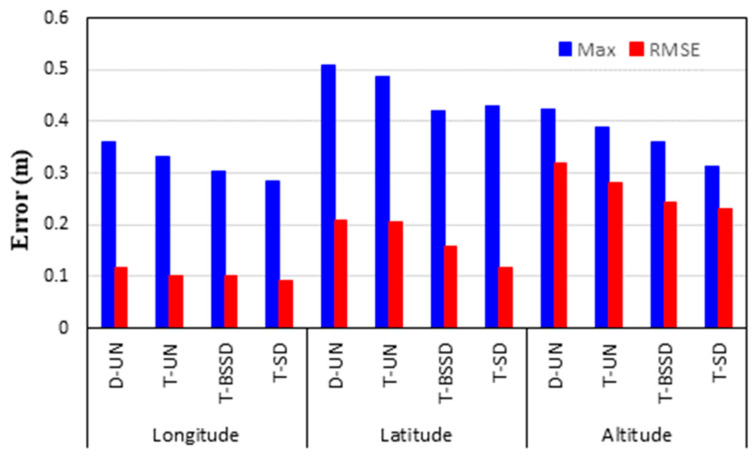
Field measurement setup for base (**left**) and rover (**right**) stations.

For kinematic application, a vehicle trajectory was implemented in Cairo on DOY 235 in 2018. Figure 2 shows the kinematic trajectory. A Leica GS15 receiver was located on board the vehicle in order to collect triple-frequency GPS/Galileo observations (Figure 3). The kinematic dataset has a 1-s time interval with a total duration of 80 min (i.e., 1.20 h). The vehicle’s coordinates were estimated using different dual- and triple-frequency kinematic PPP solutions.

For PPP processing, the IGS-MGEX precise satellite orbits and clock products were used in order to account for satellite orbit and clock errors [49]. To estimate the hydrostatic tropospheric delay, the UNB3m tropospheric model was used [50]. The float ambiguity resolution was used in our developed PPP models. For parameter estimation, the extended Kalman filter (EKF) was used. Table 2 summarizes the triple-frequency combinations that were used in our proposed PPP solutions. In addition, the combination coefficients (i.e., $\alpha_{1},\alpha_{2}$ and $\alpha_{3}$) and the noise amplification factors were computed and are also given in Table 2. The observations were processed using our developed software [18,19], which was further developed in this research in order to include processing of triple-frequency observations.

## 5. Results and Analysis

To investigate the contribution of adding the third frequency measurements to the GPS/Galileo PPP solution, various processing models were developed including undifferenced, BSSD and semi-decoupled models. The positioning accuracy and convergence time of the developed triple-frequency PPP models were validated for static and kinematic applications.

### 5.1. Validation for Static Applications

Static triple-frequency GPS/Galileo datasets were processed using four GPS/Galileo PPP models, namely, triple-frequency undifferenced PPP (T-UN), triple-frequency between-satellite single-difference PPP (T-BSSD), triple-frequency semi-decoupled PPP (T-SD) and traditional dual-frequency undifferenced ionosphere-free PPP (D-UN).

A two-hour positioning solution is selected in order to investigate the performance of the proposed GPS/Galileo PPP solutions in a short time span. For the examined three days, each day is divided into twelve sessions. Each session is processed separately for each examined station, which means that a total number of 288 sets of results are obtained.

**Figure 4:**
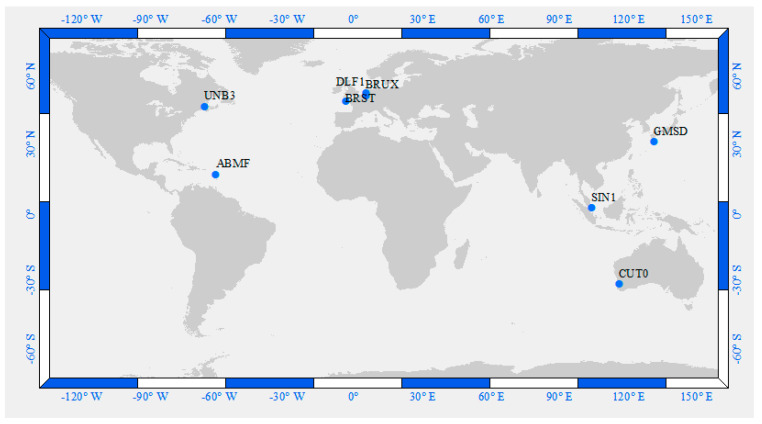
Positioning errors for stations BRST (**left**) and CUT0 (**right**) using different GPS/Galileo PPP models.

**Figure 5:**
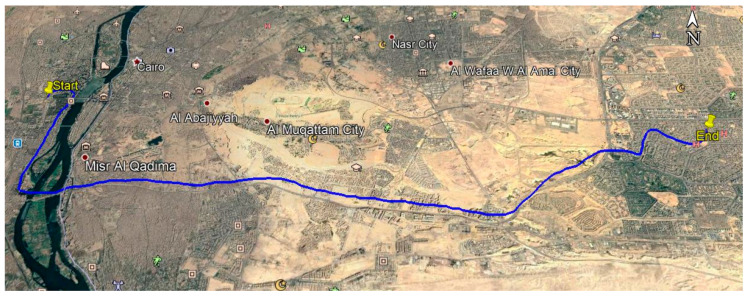
Positioning errors for stations DLF1 (**left**) and UNB3 (**right**) using different GPS/Galileo PPP models.

The positioning errors of the PPP solutions are estimated in longitude, latitude and altitude components. The four PPP solutions are referenced to the station coordinates available from the IGS [51]. Figure 4 and Figure 5 show the two-hour positioning errors in the three components for stations BRST, CUT0, DLF1 and UNB3 on DOY 1 as an example. As can be seen, the contribution of the triple-frequency observations to the convergence time is significant. Improvement in the convergence time of the triple-frequency PPP solutions is obtained in three components compared to the traditional dual-frequency PPP solution. On the other hand, comparable positioning accuracy is obtained from both dual- and triple-frequency PPP solutions at the end of the two-hour datasets.

Additionally, it can be seen that the T-SD PPP solution significantly enhances the convergence time in comparison with the other solutions. This is due to the fact that the estimation of ambiguity parameters is enhanced by separating the code and carrier phase receiver clocks. Moreover, the convergence time of T-BSSD PPP is more accelerated than the D-UN PPP and T-UN PPP solutions. This is attributed to the fact that the T-BSSD PPP model completely removes the receiver code and phase biases, which in turn improves the carrier phase ambiguities estimation. It is also shown that the T-UN PPP model speeds up the convergence time in comparison with the D-UN PPP solution. This can be attributed to the fact that the T-UN PPP model is receiver code bias-free.

**Figure 6:**
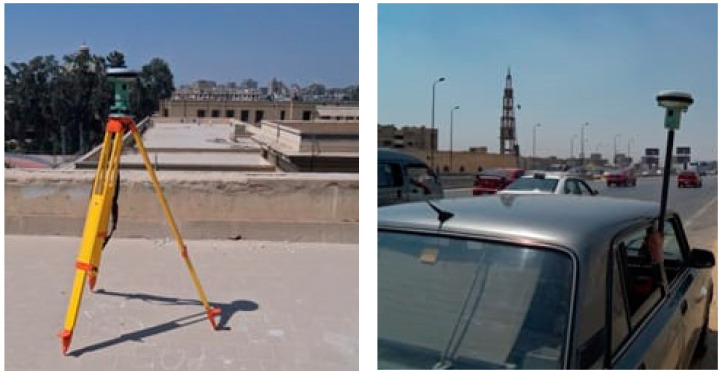
Positioning error distribution computed after 10, 20 and 60 min in longitude, latitude and altitude.

To further evaluate the performance of our developed GPS/Galileo PPP models, the positioning errors in the three components are estimated at 10, 20 and 60 min. A total number of 288 datasets are used. It should be noted that after processing every 2-h observation file, the positioning errors are computed at 10, 20 and 60 min. Figure 6 illustrates the distribution of the positioning errors for the developed PPP models computed at 10, 20 and 60 min. It is shown that the triple-frequency PPP models provide more precise solutions than the dual-frequency PPP model. In addition, comparable positioning accuracy is obtained from the three triple-frequency PPP models with superiority for the T-SD PPP model. This can be attributed to the estimation of the ambiguity parameters.

**Figure 7:**
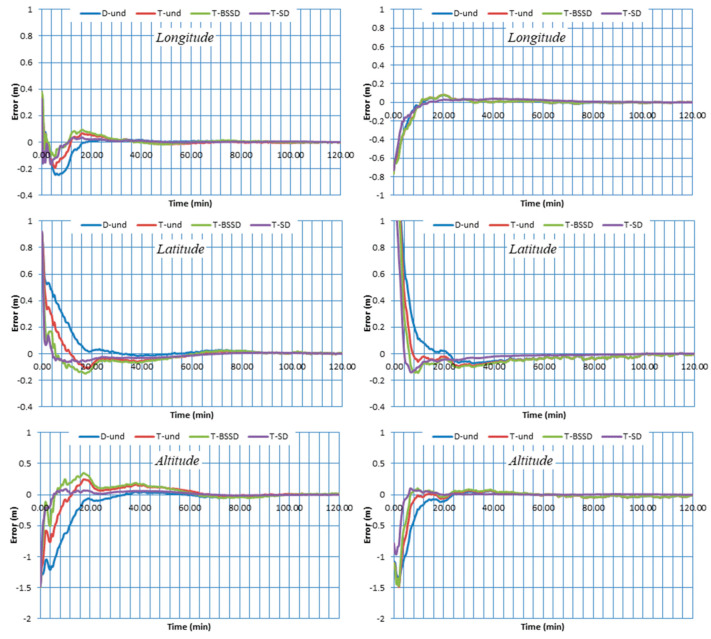
Maximum and RMSE values of the estimated positioning errors from the PPP solutions.

To further analyze the accuracy of the PPP solutions, the maximum and root mean square error (RMSE) values of the positioning errors are estimated in longitude, latitude and height components. Figure 7 shows the maximum positioning errors and the RMSE values at 10, 20 and 60 min. It is shown that both the maximum and RMSE values for the four PPP solutions are decreased with time, particularly in the height component. It is also seen that the estimation of the ambiguity parameters (i.e., T-BSSD and T-SD PPP solutions) significantly decreases the RMSE values in the three components. For the 10-min positioning, the T-SD PPP solutions are superior to the other solutions. Additionally, the DCB effect is significant on the positioning accuracy of the D-UN PPP solution. At 20 min, the positioning accuracy of both the D-UN and T-UN PPP solutions is significantly improved. In addition, slight RMSE values are obtained from both T-BSSD and T-SD PPP solutions. At 60 min, comparable positioning accuracies are obtained from the four PPP solutions. This is attributed to the fact that ambiguity parameters are fixed.

Moreover, the 3D position is determined at 10, 20 and 60 min in order to further assess the performance of our developed PPP models. Table 3 outlines the statistical parameters of the 3D position, including mean, maximum and RMSE.

It can be seen that the 3D positioning accuracy obtained through the T-SD PPP solution is enhanced by about 50, 40 and 40% after 10, 20 and 60 min, respectively, in comparison with the traditional D-UN PPP solution. In addition, the T-BSSD PPP model improves the 3D position by about 41, 31 and 40% after 10, 20 and 60 min, respectively, with respect to the D-UN PPP model. The 3D positioning accuracy obtained through the T-UN PPP model is also superior to that obtained through the D-UN PPP model by about 29, 21 and 35% at 10, 20 and 60 min, respectively.

It is also seen that the 3D positioning accuracy of each PPP model is significantly enhanced with increasing processing time, particularly for the D-UN PPP model. This is attributed to the estimation of carrier phase ambiguities. For the D-UN PPP model, the accuracy is enhanced by 0.257 and 0.363 m after 20 and 60 min, respectively, compared with 10 min. For the T-UN PPP model, the 3D position is improved by 0.17 and 0.26 m after increasing the processing time to 20 and 60 min, respectively. Furthermore, improvement in the T-BSSD PPP accuracy is obtained at 20 and 60 min by about 0.137 and 0.214 m, respectively, compared to 10 min. The 3D accuracy of the T-SD PPP model is also improved by 0.114 and 0.177 m, at 20 and 60 min, in comparison with 10 min.

In general, it can be concluded that adding the third frequency observations to the GPS/Galileo PPP solution improves the positioning accuracy in the three components after a short time span in comparison with the dual-frequency undifferenced GPS/Galileo PPP solution. Additionally, the triple-frequency PPP model provides a faster ambiguity resolution than the dual-frequency ionosphere-free PPP model.

### 5.2. Validation for Kinematic Applications

**Figure 8:**
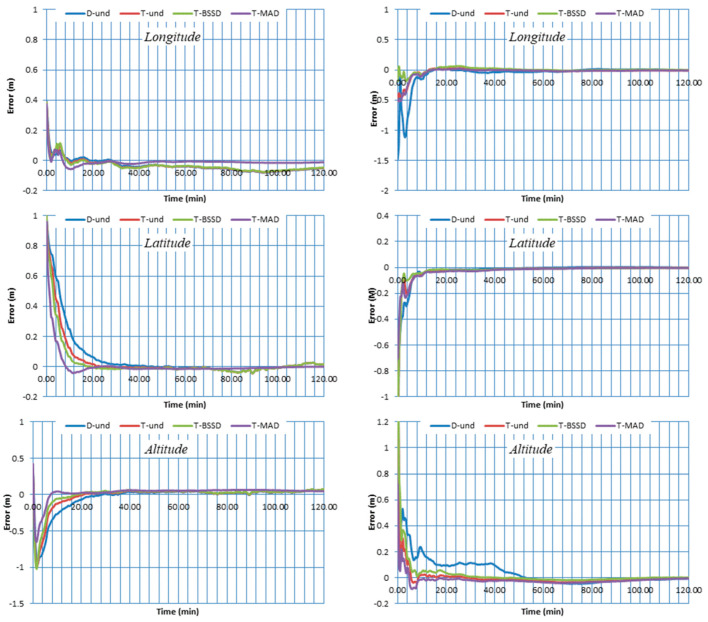
Positioning errors of different kinematic GPS/Galileo PPP solutions in longitude, latitude and altitude.

Triple-frequency GPS/Galileo observations collected from the vehicular trajectory were processed using the kinematic PPP solution. Four PPP solutions are considered, namely, dual-frequency undifferenced PPP (D-UN), triple-frequency undifferenced PPP (T-UN), triple-frequency BSSD PPP (T-BSSD) and triple-frequency semi-decoupled PPP (T-SD). The traditional relative GNSS solution is used as a reference. The positioning errors of the four kinematic PPP solutions are estimated in longitude, latitude and altitude components and then illustrated in Figure 8.

As can be seen from Figure 8, the kinematic T-BSSD PPP and T-SD PPP solutions slightly converged before the D-UN PPP and T-UN PPP solutions. This is due to the improvement in ambiguity estimations, which resulted from elimination of receiver code and phase biases, and separation of receiver clock code and phase clocks for T-BSSD PPP and T-SD PPP solutions, respectively. Additionally, the effect of the receiver code bias on the convergence time is clear for the D-UN PPP solution.

**Figure 9:**
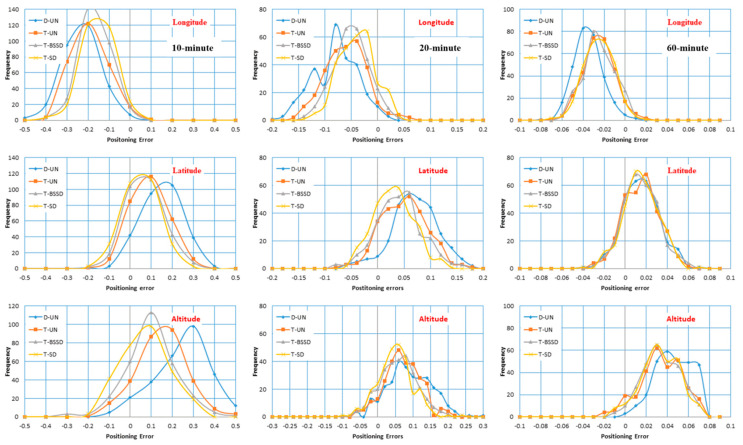
Maximum and RMSE values of the positioning errors for different kinematic PPP solutions.

To analyze the positioning accuracy of the kinematic PPP solutions, the differences between the coordinates obtained through the kinematic PPP solutions and those obtained through the relative solution are computed. The maximum and RMSE values for the differences are then computed. Figure 9 illustrates the maximum and STD values for the differences in longitude, latitude and altitude components.

It is shown that the maximum values of the positioning error are less than 0.36, 0.51 and 0.42 m in longitude, latitude and height components, respectively. It can also be seen that the positioning accuracy is improved in the three components when the triple-frequency PPP models are used, particularly the T-SD model. For the longitude component, the RMSEs are decreased slightly by 0.01, 0.02 and 0.03 m for the T-UN, T-BSSD and T-SD PPP models, respectively, in comparison with the D-UN PPP model. On the other hand, a significant reduction can be noticed in both latitude and height components. For the latitude component, the RMSE values are reduced by 0.01, 0.06 and 0.11 m for the T-UN, T-BSSD and T-SD PPP models, respectively, with respect to the D-UN PPP model. The error in the height component is decreased by 0.04, 0.08 and 0.09 m when the T-UN, T-BSSD and T-SD PPP models are used, respectively, compared to the D-UN PPP model.

The positioning accuracy of the proposed kinematic PPP solutions are further evaluated through computation of the mean, maximum and RMSE of the 3D positioning (Table 4). It is found that the 3D position of the T-SD PPP solution is enhanced by 31% in comparison with the D-UN PPP solution. In addition, the T-BSSD PPP solution improves the 3D positioning accuracy by about 23% with respect to the D-UN PPP solution. Improvement in the positioning accuracy by about 10% is obtained when the T-UN PPP solution is used compared to the D-UN PPP solution.

Based on the obtained results from static and kinematic positioning, it can be seen that the non-integer ambiguity parameter, which contains the code biases for both the satellite and receiver, has a significant impact on the convergence time of the traditional undifferenced dual-frequency PPP solution. The undifferenced triple-frequency PPP solution, however, is free of receiver code bias. Thus, it converged faster than the undifferenced dual-frequency solution. In addition, removing the receiver code and phase biases from triple-frequency observations in the BSSD PPP solution speeds up the convergence time compared to the undifferenced triple-frequency PPP solution. A faster convergence time is obtained when the triple-frequency semi-decoupled PPP solution is used as the code and phase clocks are separated. After processing the 2-h datasets, comparable positioning accuracy is obtained from both dual- and triple-frequency PPP solutions. This is attributed to the fact that the EKF estimates used the parameters in an efficient way. On the other hand, much faster convergence can be obtained if the ambiguity resolution solution (i.e., PPP-AR) is used.

## 6. Conclusions

In this paper, new PPP models were developed by combining triple-frequency GPS/Galileo observations. Different triple-frequency PPP techniques were used including undifferenced, BSSD and semi-decoupled. The traditional dual-frequency undifferenced PPP solution was also used. The proposed models were validated for static and kinematic positioning applications. Static triple-frequency GPS/Galileo observations from eight globally distributed reference stations spanning three successive days were used. Compared with the dual-frequency PPP solution, the 3D position was improved after 10 min by about 50, 41 and 29% for the semi-decoupled, BSSD and undifferenced PPP solutions, respectively, and it was also enhanced after 20 min by about 40, 31 and 21% for the semi-decoupled, BSSD and undifferenced PPP solutions. Further 3D position improvement was acquired after 60 min by about 40, 40 and 35% for the semi-decoupled, BSSD and undifferenced PPP solutions, respectively, in comparison with the dual-frequency solution. Moreover, triple-frequency GPS/Galileo observations from a vehicle trajectory were processed using the four proposed PPP solutions. It was found that the 3D positioning accuracy of the semi-decoupled, BSSD and undifferenced PPP solutions was enhanced by about 31, 23 and 10% with respect to the dual-frequency PPP solutions. It can be concluded that adding the triple-frequency observations to the PPP solution accelerates the convergence time and improves the positioning accuracy in comparison with the traditional dual-frequency PPP solution. In addition, the convergence time and positioning accuracy of our developed triple-frequency semi-decoupled PPP model are superior compared to the other developed PPP models, which can be used in precise surveying, deformation monitoring and hydrographic applications.

## Figures and Tables

**Table 1:** Properties of examined stations.

Station	Longitude	Latitude	Receiver
BRST	−4.497°	48.38°	Trimble Alloy
GMSD	131.016°	30,556°	Trimble NETR 8
UNB3	−66.642°	45.95°	Trimble Alloy
CUT0	115.895°	−32.004°	Trimble NETR 8
DLF1	4.387°	51.986°	Trimble NETR 9
ABMF	−61.528°	16.262°	Trimble NETR 10
SIN1	103.679°	1.343°	Trimble NETR 11
BRUX	4.359°	50.798°	SEPT POLARX5TR

**Table 2:** Triple-frequency combinations, their coefficients and noise amplification factors [40].

Constellation	Combination	$\alpha_{1}$	$\alpha_{2}$	$\alpha_{3}$	Noise Factor
GPS	L1/L2/L5	2.327	−0.360	−0.968	2.546
Galileo	E1/E5a/E5b	2.315	−0.836	−0.479	2.507

**Table 3:** Statistical parameters of 3D position accuracy for different PPP solutions (in meters).

PPP Solution	10 min			20 min			60 min		
	Mean	Max	RMSE	Mean	Max	RMSE	Mean	Max	RMSE
D-UN	0.403	0.901	0.406	0.146	0.385	0.149	0.037	0.092	0.043
T-UN	0.281	0.841	0.288	0.112	0.359	0.118	0.013	0.083	0.028
T-BSSD	0.226	0.761	0.240	0.095	0.315	0.103	0.001	0.093	0.026
T-SD	0.185	0.614	0.203	0.078	0.304	0.089	0.001	0.094	0.026

**Table 4:** Statistical parameters of 3D position accuracy for different kinematic PPP solutions.

PPP Solution	Statistical Parameter (m)		
	Mean	Max	RMSE
D-UN	0.351	0.754	0.400
T-UN	0.327	0.706	0.361
T-BSSD	0.250	0.631	0.308
T-SD	0.184	0.603	0.275

## Data Availability

The data presented in this study are available on request from the corresponding author. The data are not publicly available due to technical secrets.
